# Kinetics of Plasma Cytokines, Angiopoietin-2, and C-Reactive Protein in Dogs With Gastric Dilatation Volvulus

**DOI:** 10.3389/fvets.2021.652479

**Published:** 2021-06-16

**Authors:** Anna Brunner, Simone Schuller, Bianca Hettlich, Eliane Marti, Anna Lehmann, Laureen M. Peters, Katja-Nicole Adamik

**Affiliations:** ^1^Division of Small Animal Surgery, Department of Clinical Veterinary Science, Vetsuisse Faculty, University of Bern, Bern, Switzerland; ^2^Division of Small Animal Internal medicine, Department of Clinical Veterinary Science, Vetsuisse Faculty, University of Bern, Bern, Switzerland; ^3^Department of Clinical Research and Veterinary Public Health, Vetsuisse Faculty, University of Bern, Bern, Switzerland; ^4^Clinical Diagnostic Laboratory, Department of Clinical Veterinary Science, Vetsuisse Faculty, University of Bern, Bern, Switzerland; ^5^Division of Small Animal Emergency and Critical Care, Department of Clinical Veterinary Science, Vetsuisse Faculty, University of Bern, Bern, Switzerland

**Keywords:** inflammatory markers, acute-phase protein, canine gastric torsion, ischemia-reperfusion, SIRS

## Abstract

**Background:** The degree of systemic inflammation, reperfusion injury and endothelial activation are potentially important determinants of clinical outcomes in dogs with gastric dilatation volvulus (GDV).

**Objective:** To evaluate plasma concentrations and kinetics of inflammatory markers in dogs with GDV over a time frame of 48 h, and to compare to healthy dogs.

**Design and Setting:** Prospective, observational cohort study in client-owned dogs with GDV.

**Materials and Methods:** Fifteen dogs with GDV and 9 healthy control dogs were enrolled. Plasma concentrations of interleukin (IL)-6, IL-7, IL-8, IL-10, IL-15, IL-18, interferon gamma (IFN-γ), keratinocyte chemotactic-like, monocyte chemotactic protein (MCP)-1, Angiopoietin (Ang)-2, and C-reactive protein (CRP) were measured at admission (prior any therapeutic intervention, (T0), immediately after surgery (T1), 24 ± 4 h (T24), and 48 ± 4 h (T48) post-surgery. Cytokines were measured using multiplex magnetic bead assay. Plasma Ang-2 was measured with a commercial human ELISA test kit validated for dogs.

**Results:** Dogs with GDV had significantly higher plasma concentrations of IFN-γ and IL-10 compared to healthy control dogs at all time points. Concentrations of IL-6 were significantly higher at T1 and T24, concentrations of MCP-1 at T24, and concentrations of CRP at T24 and T48. A significant increase between T0 and T1 was found for IL-6, IL-10, and CRP, between T1 and T24 for IL-8, IFN-γ, MCP-1, and CRP, and between T24 and T48 for IL-15, Ang-2, and CRP. A significant decrease between T0 and T1 was found for IL-7, IL-8, IL-15, IL-18, and Ang-2; between T1 and T24 for IL-6 and KC-like; and between T24 and T48 for IL-6.

**Conclusion:** In GDV dogs, a mild pro-inflammatory reaction was present at admission, which peaked immediately after and up to 24 h post-surgery, mainly represented by IL-6, IFN-γ, MCP-1, and CRP, and which decreased at T48. In addition, the anti-inflammatory IL-10 was increased in GDV dogs at all time points.

## Introduction

Canine gastric dilatation volvulus (GDV) is a life-threatening condition characterized by gastric displacement and cardiovascular shock. Subsequent systemic hypoperfusion, gastric ischemia and reperfusion, gastric wall necrosis, and gastro-intestinal bacterial translocation trigger an inflammatory response leading to a systemic inflammatory response syndrome (SIRS) (1). Triggers of this sterile inflammation are damage-associated molecular patterns (e.g., molecules released from necrotic cells) but also microbial pathogen-associated molecular patterns (2). In addition, intracellular stores of pro-inflammatory cytokines and chemokines are released by necrotic cells (2). In the context of GDV, medical interventions with the aim to restore systemic and local perfusion, such as intravenous (IV) fluid therapy and surgical gastric repositioning, paradoxically can aggravate injury through multiple mechanisms, recognized as ischemia-reperfusion injury (IRI). The cascade of IRI involves activation of neutrophils, platelets, cytokines, reactive nitrogen species, reactive oxygen species (ROS), the coagulation system, the endothelium, and the xanthine-oxido-reductase enzyme system (3). All these factors may potentially lead to irreversible organ damage and failure (1, 3, 4).

Only few studies have evaluated inflammatory parameters in dogs with GDV (5–7). One reported high mobility group box-1 (HMGB-1) to be prognostic in dogs with GDV, with higher baseline and post-surgery concentrations in non-survivors (7). In another study, concentrations of cell free DNA (cfDNA), HMGB-1, and procalcitonin (PCT) were increased in dogs with GDV, but only PCT concentrations were predictive of non-survival (6). Cytokines play multiple, integral roles in the immune defense against infection and neoplasia, but they are also mediators of inflammatory diseases (8, 9). By using multiplex cytokine analysis technique (8), cytokine profiles have been determined in numerous diseases in dogs, such as immune-mediated hemolytic anemia (IMHA) (10, 11), critical illness and sepsis (12, 13), pyometra (14, 15), and trauma (16), amongst others. Data on the cytokine profile in dogs with GDV are lacking. Angiopoietin-2 (Ang-2) plays a role in regulating angiogenesis and inflammation and is an important player in the regulation of vascular endothelial activation and dysfunction (17, 18). Plasma concentrations of Ang-2 are increased in dogs with SIRS and sepsis and are correlated with disease severity and outcome (19). In critically ill people with SIRS elevated plasma concentrations of Ang-2 are associated with death and organ dysfunction (20). No study has yet evaluated Ang-2 concentrations in dogs with GDV. C-reactive protein (CRP) is a major acute phase protein in dogs, and its synthesis in the liver is stimulated by pro-inflammatory cytokines (e.g., Interleukin (IL)-6). Serum concentrations increase in dogs with systemic inflammation following surgery, trauma, infections, or neoplasia (21). Concentrations of CRP were commonly increased in dogs with GDV (7, 22, 23) and were significantly associated with a negative outcome in one study (24).

The objective of this study was to quantify the inflammatory state of dogs with GDV by evaluating plasma concentrations of different cytokines, Ang-2, and CRP over a period of 48 h, and by comparing cytokines and CRP to values of healthy control dogs. Our hypotheses were that (1) admission concentrations of inflammatory biomarkers are higher in dogs with GDV compared to healthy dogs, that (2) concentrations of biomarkers rise after surgery and repositioning of the stomach, and that (3) concentrations decrease again by 48 h after surgery.

## Materials and Methods

### Animals

This was a prospective study including 15 client-owned dogs with GDV and 9 healthy staff-owned control dogs. The study was approved by the Animal Experiment Committee of the Swiss Federal Veterinary Office (registration number BE 69/17), and informed owner consent was obtained for all dogs prior to inclusion. Dogs with GDV were enrolled between June 2017 and September 2018 at the Small Animal Clinic, Vetsuisse Faculty, University of Bern, Switzerland. Diagnosis of GDV was based on compatible clinical signs and confirmed by characteristic findings of a right lateral abdominal radiograph and during surgical abdominal exploration. Dogs receiving IV lidocaine (e.g., with cardiac arrhythmias necessitating treatment with lidocaine) were excluded throughout due to the potential anti-inflammatory effect of this drug. Dogs that were euthanized during the study period due to financial reasons were also excluded.

Control dogs were classified as healthy based on an uneventful medical history, physical examination, and unremarkable complete blood count and serum chemistry results. Dogs were eligible if they had no history or evidence of recent or chronic medical conditions and had not received any medication, except for routine preventative healthcare, within the preceding 3 months.

### Severity of Illness Scores

In GDV dogs, an acute patient physiologic and laboratory evaluation (APPLE_fast_) (25) score was calculated for each dog at admission. The APPLE_fast_ score (ranging from 0 [predicted mortality 0%] to 50) is a 5-variable, diagnosis independent, severity-of-illness score based on plasma concentrations of albumin and glucose, thrombocyte concentration, blood lactate concentration, and mentation score (25). The presence or absence of SIRS was determined at admission and at 24 h after surgery by evaluating heart rate, respiratory rate, white blood cell concentration, and rectal temperature. Dogs were considered to have SIRS when ≥2 of the established SIRS criteria (hypothermia or hyperthermia, leukocytosis or leukopenia, tachycardia, and tachypnea) were fulfilled (26). Survival was defined as discharge from the hospital. Death was defined as naturally deceased or euthanasia before hospital discharge.

### Cardiovascular Stabilization

All GDV dogs underwent a standardized stabilization protocol. For cardiovascular stabilization, supplemental oxygen and IV isotonic balanced and buffered crystalloids (Plasma-Lyte A^®^, Baxter AG, Switzerland) was administered at the clinician's discretion. Total administered fluid volumes were recorded throughout the study period. Initial analgesia was provided by either IV methadone (0.2 mg/kg; Methadon Streuli^®^, Streuli Pharma AG, Switzerland), or a bolus of fentanyl (5 μg/kg; Fentanyl Curamed^®^, Actavis Switzerland AG, Switzerland) followed by a continuous rate infusion (CRI) of fentanyl at a rate of 5 μg/kg/h. In dogs with severe gastric distension, transcutaneous gastrocentesis for gastric decompression with a 14- or 16-gauge needle was performed after initiation of fluid therapy.

### Anesthesia, Surgery, and Post-Operative Monitoring

A standardized anesthesia protocol was used for all GDV dogs. Premedication was initiated with IV methadone (0.2 mg/kg) and anesthesia was induced with IV midazolam (0.2 mg/kg; Dormicum^®^, Roche Pharma SA, Switzerland) and propofol (to effect; Propofol-^®^Lipuro, B. Braun Medical AG, Switzerland). After endotracheal intubation, anesthesia was maintained with isoflurane (titrated to effect; Isoflo^®^ ad us. vet., Zoetis GmbH, Switzerland) and oxygen (60–100%). Analgesia was provided by IV fentanyl CRI at a rate of 5 μg/kg/h. During anesthesia, continuous electrocardiogram (ECG), capnography, pulsoxymetry, arterial BP, and esophageal temperature were monitored.

Exploratory laparotomy was performed by a board-certified surgeon or a senior surgery resident and included decompression and repositioning of the stomach and subsequent gastropexy. Gastric wall changes were classified based on gross appearance of the stomach after repositioning and divided into mild (no or slight red coloration of the stomach wall), moderate (purple or hemorrhaged gastric wall) and severe (green, gray or black gastric wall color and a friable and palpably thin gastric wall).

Postoperative monitoring included heart rate and cardiac rhythm, mucous membrane color and capillary refill time, mentation, respiratory rate, rectal temperature, and oscillometric BP. A continuous ECG was monitored during the first 12–24 h post-surgery, and subsequent intermittent ECG was performed every 8 h until 48 ± 4 h post-surgery. Intravenous fentanyl was administered up to 24 h post-surgery and then replaced by IV buprenorphine (0.01–0.02 mg/kg q8h; Temgesic^®^, Indivior Schweiz AG). If there was a clinical indication, fentanyl CRI was stopped earlier than 24 h post-surgery. Supportive treatment included IV isotonic crystalloids to maintain intravascular volume and hydration, as well as omeprazole (1 mg/kg IV q12h; Omeprazol Streuli^®^, Streuli Pharma AG, Switzerland).

### Blood Sample Processing and Analyses of Inflammatory Markers

Venous blood samples were obtained at four different time points, namely at admission (prior to any therapeutic intervention, T0), in the immediate postsurgical period (T1), 24 ± 4 h (T24), and 48 ± 4 h (T48) post-surgery. At T0, T24, and T48, blood was collected in a 1.3 ml K2-EDTA tube and a 9 ml heparin tube (K2-EDTA and Li-Heparin LH/1.3, Sarstedt AG, Switzerland) for analyses of hematological (Advia^®^ 2120i, Siemens Healthcare Diagnostics AG, Switzerland) and biochemical variables (Cobas^®^ c501, Roche Diagnostics, Switzerland), CRP (Randox canine CRP, CP2798, Cobas^®^ c501, Roche Diagnostics, Switzerland), and lactate (RAPIDPoint^®^ 500; Siemens Healthcare AG, Switzerland), necessary for calculating the APPLE_fast_ score and diagnosing SIRS. At T1, only blood for the 9 ml heparin tube was taken. After lactate analysis, heparinized blood was centrifuged (3,000 RPM, 20°C for 10 min). An aliquot (0.5 ml) of the heparinized plasma was used for the biochemical and CRP analysis. The remaining plasma was aliquoted and placed in a −80°C freezer within 1 h of blood collection until batch analyses of cytokines and Ang-2 was performed. For dogs euthanized intraoperatively, the T1 blood sample was collected after gastric repositioning, immediately prior to euthanasia.

Cytokine and Ang-2 measurements were performed by a trained laboratory technician between February and April 2019. Cytokines were analyzed in duplicate using a Milliplex Canine Cytokine Panel, CCYTOMAG-90K kit (Luminex MAGPIX analyzer, EMD Millipore, USA), which is described in detail elsewhere (8). The kit was used according to the manufacturer's instructions with an overnight incubation at 4°C on a plate shaker. Based on pilot data collected by the authors of this study, concentrations of tumor necrosis factor-α and IL-2 were below the level of detection in 40 plasma samples of GDV dogs and were therefore not analyzed in our study. The following cytokines were analyzed: IL-6, IL-7, IL-8, IL-10, IL-15, IL-18, interferon gamma (IFN-γ), keratinocyte chemotactic-like (KC-like), and monocyte chemotactic protein (MCP-1). The measurements were performed in accordance with the manufacturer's instructions with internal quality control. Samples were randomized on each plate. Cytokine concentrations were given in pg/ml.

For determination of plasma Ang-2 concentrations, a commercial human ELISA test kit (Human Angiopoietin 2 Immunoassay; R&D Systems, Minneapolis, Minnesota, USA) was used, which has been validated for the use in dogs (27). The concentrations were given in pg/ml.

In the healthy control dogs, concentrations of plasma cytokines and CRP were analyzed at a single time point. Due to limited aliquot size, Ang-2 concentrations could not be determined in this group.

### Statistical Analyses

All statistical analyses were performed using a commercial software (MedCalc Software Ltd B-8400 Ostend, Belgium). Descriptive statistics (frequency table and summary statistics table) were calculated for clinical and laboratory analyses. A Fisher's exact test was used to assess differences between groups for categorical clinical data namely sex and SIRS. Due to small group size, cases with moderate and severe gastric wall changes were grouped together for statistical analyses. As some data were normally distributed but others were not and could not be transformed to normality, non-parametric analyses were performed. A Mann-Whitney test for continuous data (age, weight, APPLE_fast_ score, CRP, lactate, Ang-2, IL-6, IL-7, IL-8, IL-10, IL-15, IL-18, IFN-γ, KC-like, MCP-1, and CRP) was used. When the cytokine concentrations were below the limit of detection, the result was recorded as “0.” For within-group and between-time comparison, a Wilcox signed rank test was performed. To test possible correlations between variables Spearman's coefficient of rank correlation (rho) with 95% confidence interval was used. Significance was set at ≤0.05 throughout.

## Results

### Healthy Control Dogs

Of the 9 control dogs, 4 were male (3 castrated, 1 intact) and 5 were female (2 spayed, 3 intact) ranging in age from 1.8 to 10.5 years (mean, 6.6 years). Breeds represented were German Shepherd (*n* = 4), and 1 each of Labrador Retriever, Golden Retriever, St. Bernard, Belgian Tervuren Dog, and mixed breed. The median body weight was 34 kg (range, 28–81 kg). No significant difference was found for body weight and age between GDV and control dogs.

### GDV Dogs

Of the 15 dogs with GDV, 8 were male (4 castrated, 4 intact) and 7 were female (6 spayed, 1 intact) ranging in age from 2.1 to 14.5 years (mean, 8.2 years). Breeds represented were mixed breed (*n* = 5), St. Bernard (*n* = 2), Weimaraner (*n* = 2), and 1 each of Great Dane, Golden Retriever, Labrador Retriever, Spanish Mastiff, Newfoundland dog, and Standard Poodle. Median body weight was 35 kg (range, 17–87 kg). Median duration of clinical signs until presentation was 120 min (range, 60–360 min).

The median heart rate at admission was 140 bpm (range, 88–210 bpm), median rectal temperature was 38.4°C (range, 37.8–39.5°C), and median respiratory rate was 44 rpm (range, 20–120 rpm). At T0, 12/15 dogs (80%) and at T2, 8/12 dogs (67%) were classified as having SIRS. The median APPLE_fast_ score at T0 was 20 (range, 10–34). Clinical parameters, presence of SIRS, and APPLE_fast_ scores for T0, T1, T24, and T48 are presented in Table 1. Transcutaneous gastrocentesis for gastric decompression during cardiovascular stabilization was performed in 12/15 dogs.

**Table 1:** Clinical parameters, SIRS, and APPLE_fast_ scores at admission (T0), post-surgery (T1), 24 h (T24), and 48 h (T48) post-surgery in dogs with GDV.

**Variable**	**Time point**	**Values in GDV dogs**
Heart rate (bpm)	T0	140 (88–210)
	T1	94 (72–160)
	T24	82 (72–120)
	T48	80 (64–100)
Rectal temperature (°C)	T0	38.4 (37.8–39.5)
	T1	37.4 (36.6–38.1)
	T24	38.0 (37.2–39.4)
	T48	38.1 (37.4–38.8)
Respiratory rate (rpm)	T0	44 (20–120)
	T1	24 (20–80)
	T24	28 (20–69)
	T48	36 (20–80)
Lactate (RI:0.43–2.10 mmol/L)	T0	2.77 (1.35–10.38)
	T1	1.34 (0.55–3.21)
	T24	1.11 (0.75–1.71)
	T48	0.91 (0.62–2.45)
Presence of SIRS	T0	12 of 15 dogs
	T1	n/a
	T24	8 of 12 dogs
	T48	n/a
APPLE_fast_ score	T0	20 (10–34)
	T1	n/a
	T24	21 (15–23)
	T48	20 (15–25)

*Values are presented as median (min-max); number of dogs at T0 and T1 = 15; number of dogs at T24 and T48 = 12; bpm, beats per minute; rpm, breaths per minute; RI, reference interval; SIRS, systemic inflammatory response syndrome; APPLE, acute patient physiologic and laboratory evaluation; n/a, not applicable*.

All dogs had evidence of gastric wall changes during surgery, judged to be mild (*n* = 10), moderate (*n* = 2), or severe (*n* = 3). None of the dogs underwent partial gastrectomy. Overall mortality rate was 20% (3/15). One dog (with severe gastric wall changes) was euthanized intraoperatively and two dogs (one with moderate and one with severe gastric wall changes) experienced cardiopulmonary arrest unresponsive to cardiopulmonary resuscitation at 3- and 6-h post-surgery, respectively. The other 12 dogs recovered well and were discharged. Mean length of hospitalization was 2.5 days (range, 2–3.5). The APPLE_fast_ score at T0 in the 5 dogs with moderate/severe gastric wall changes (median 29; range, 15–34) was significantly higher compared to the 10 dogs with mild gastric wall changes (median 15; range, 10–26) (*P* = 0.017). The median blood lactate concentration at admission was 2.8 mmol/L (range, 1.4–10.4 mmol/L) (Table 1). Dogs with moderate/severe gastric wall changes had significantly higher median lactate concentrations (8.4 mmol/L; range, 2.1–10.4 mmol/L) compared to dogs with mild gastric wall changes (2.0 mmol/L; range, 1.4–3.4 mmol/L) (*P* = 0.010) (Figure 2D). Median lactate concentration in non-survivors was 8.5 mmol/L (range, 4.8–10.4) and in survivors 2.0 mmol/L (range, 1.4–8.4 mmol/L). No statistical analysis was performed due to small sample size of the non-survivor group (*n* = 3).

The median administered fluid volumes between T0 and T1 were 81 ml/kg (range, 52–170 ml/kg); between T1 and T24, 58 ml/kg (range, 42–101 ml/kg); and between T24 and T48, 48 ml/kg (range, 4–89 ml/kg). The median time between admission and start of the surgery was 100 min (range, 65–205 min). Median time of surgery was 65 min (range, 40–100 min).

### Inflammatory Markers

**Figure 1:**
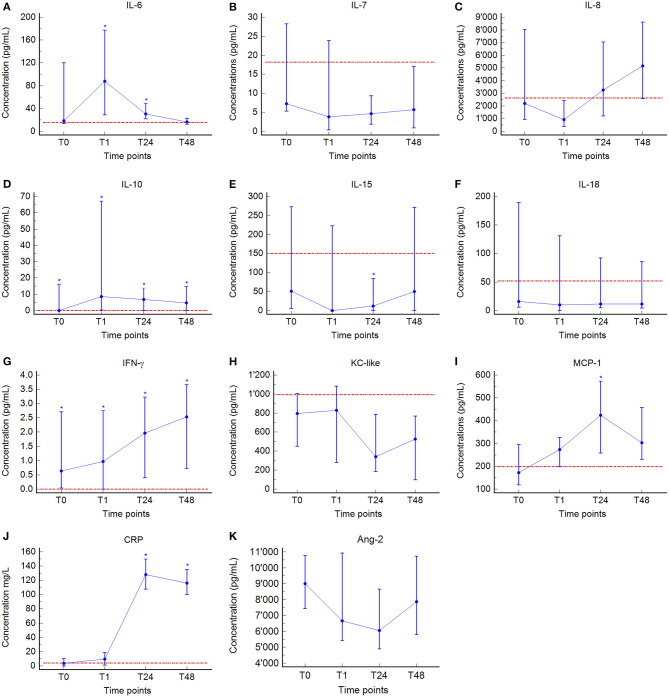
Median (blue central value marker-dot) and interquartile range (blue error bars) of plasma concentrations of Interleukin (IL)-6 **(A)**, IL-7 **(B)**, IL-8 **(C)**, IL-10 **(D)**, IL-15 **(E)**, IL-18 **(F)**, interferon (IFN)-γ **(G)**, keratinocyte chemotactic-like (KC-like) **(H)**, monocyte chemotactic protein (MCP)-1 **(I)**, C-reactive protein (CRP) **(J)**, and angiopeietin-2 (Ang-2) **(K)** at admission (prior any therapeutic intervention, T0), immediately after surgery (T1), 24 h (T24), and 48 h (T48) post-surgery. Red dotted line, median of plasma concentrations of the control group. ^*^indicates significant difference (*P* < 0.05) between GDV and control dogs (Mann-Whitney test). No control group-values were available for Ang-2.

Median values and interquartile ranges of the cytokine concentrations are presented in Table 2 and Figures 1A–K. In dogs with GDV, significantly higher plasma concentrations of IL-10 (*P* = 0.029) and IFN-γ (*P* = 0.014) at T0, of IL-6 (*P* = 0.013), IL-10 (*P* = 0.002), and IFN-γ (*P* = 0.036) at T1; of IL-6 (*P* = 0.039), IL-10 (*P* = 0.007), IFN-γ (*P* = 0.002), MCP-1 (*P* = 0.023), and CRP (*P* < 0.001) at T24, and of IL-10 (*P* = 0.007), IFN-γ (*P* = 0.002), and CRP (*P* < 0.001) at T48 were found compared to the healthy controls (Table 2, Figure 1). Significantly lower concentrations were found in GDV dogs compared to healthy dogs for IL-15 at T24 (*P* = 0.048) (Table 2, Figure 1). No significant differences between GDV and healthy dogs were found for IL-7, IL-8, IL-18, and KC-like.

**Table 2:** Median (interquartile range) concentrations of inflammatory markers at admission (T0), post-surgery (T1), 24 h (T24), and 48 h (T48) post-surgery in dogs with gastric dilatation volvulus (GDV) and in healthy control dogs.

**Parameter**	**Healthy control**	**GDV dogs**			
	**(*n* = 9)**	**T0 (*n* = 15)**	**T1 (*n* = 15)**	**T24 (*n* = 12)**	**T48 (*n* = 12)**
IL-6 (pg/ml)	15.6 (0–23.0)	18.6 (13.4–120)	88.0 (28.9–179)^*§^	30.4 (21.7–48.7)^*§^	16.6 (12.7–22.9)^§^
IL-7 (pg/ml)	18.2 (0–50.1)	7.3 (5.3–28.3)	3.8 (0.4–23.9)^§^	4.6 (1.8–9.3)	5.7 (0.9–17.1)
IL-8 (pg/ml)	2627 (1135–4382)	2192 (941–8045)	910 (372–2444)^§^	3247 (1219–7056)^§^	5157 (2573–8613)
IL-10 (pg/ml)	0 (0–0)	0.0 (0.0–16.2)^*^	8.5 (0.4–67.2)^*§^	6.9 (0.0–13.9)^*^	4.8 (0.0–14.8)^*^
IL-15 (pg/ml)	150 (90.0–608)	50.5 (5.3–273)	0.9 (0.0–223)^§^	12.1 (0.0–84.6)^*^	50.5 (0.0–272)^§^
IL-18 (pg/ml)	52.1 (0–83.6)	16.0 (5.9–190)	9.8 (0.0–131)^§^	11.5 (4.9–93.3)	11.7 (4.6–85.7)
IFN-γ (pg/ml)	0 (0–0)	0.6 (0.04–2.7)^*^	0.97 (0.00–2.8)^*^	1.97 (0.4–3.2)^*§^	2.5 (0.7–3.7)^*^
KC-like (pg/ml)	994 (326–1087)	797 (452–1007)	829 (282–1085)	342 (186–787)^§^	516 (98.2–770)
MCP-1 (pg/ml)	199 (166–306)	172 (119–296)	273 (200–327)	424 (258–574)^*§^	303 (231–458)
Ang-2 (pg/ml)	n/a	9006 (7438–10792)	6669 (5425–10936)^§^	6058 (4896–8652)	7871 (5792–10733)^§^
CRP (mg/L)	3.8 (1.1–5.2)	3.4 (0.2–10.4)	9.5 (1.2–18.8) ^§^	128 (108–150)^*§^	116 (100–135)^*§^

Legend: within the same line, a significant difference from the previous time is denoted with a paragraph (§ = P < 0.05); a significant difference from the control group is denoted with asterisk (^*^ = P < 0.05). exact P-values are provided in the result section; n/a, not applicable.

Over time, a significant increase between T0 and T1 was found for IL-6 (*P* = 0.020), IL-10 (*P* = 0.001), and CRP (*P* = 0.005); between T1 and T24 for IL-8 (*P* = 0.001), IFN-γ (*P* = 0.012), MCP-1 (*P* = 0.021), and CRP (*P* = 0.0002); and between T24 and T48 for IL-15 (*P* = 0.039), Ang-2 (*P* = 0.009), and CRP (*P* = 0.009) (Table 2, Figure 1). A significant decrease between T0 and T1 was found for IL-7 (*P* = 0.001), IL-8 (*P* = 0.001), IL-15 (*P* = 0.001), IL-18 (*P* = 0.001), and Ang-2 (*P* = 0.002); between T1 and T24 for IL-6 (*P* = 0.016) and KC-like (*P* = 0.012); and between T24 and T48 for IL-6 (*P* = 0.001) (Table 2, Figure 1). Duration of clinical signs in GDV dogs did not correlate to the concentration of any cytokine at T0 (data not shown).

**Figure 2:**
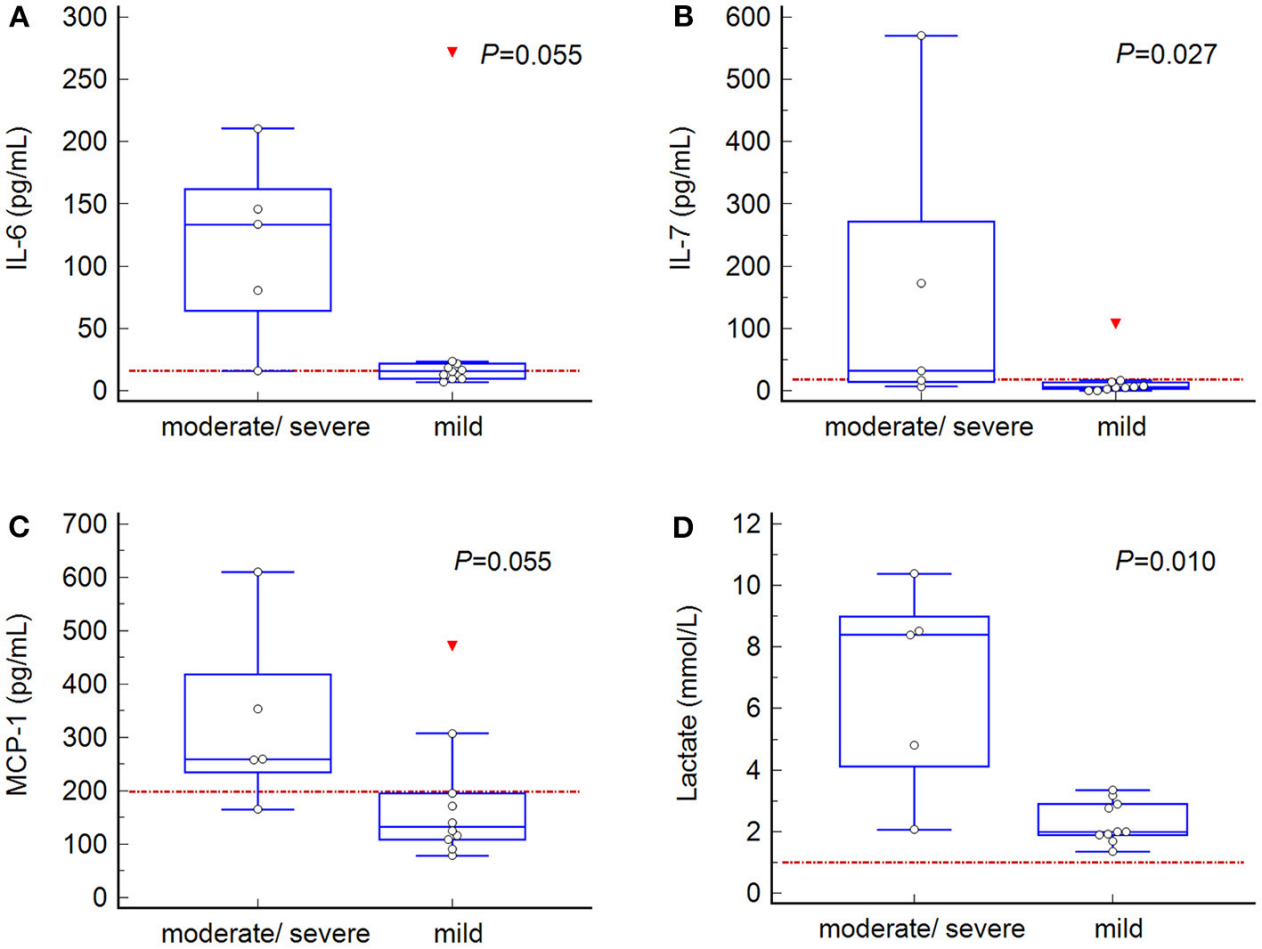
Box-and-whisker plots of admission blood concentrations of markers, in which significant or borderline significant differences were found between values from dogs with mild (*n* = 10) vs. moderate/severe (*n* = 5) gastric wall changes. **(A)** interleukin (IL)-6; **(B)** IL-7; **(C)** monocyte chemotactic protein (MCP)-1; **(D)** lactate. Central box, values from the lower to upper quartile (25-75 percentile); middle line, median; horizontal line extends from the minimum to the maximum value; each displayed point represents the value of one dog; the red triangle represents an outlier. Red dotted line, median value of the control dogs. The *P*-value represents the statistical difference between dogs with mild versus moderate/severe gastric wall changes (Mann Whitney test).

Comparison of admission plasma cytokine concentrations between dogs with mild (*n* = 10) vs. moderate/severe (*n* = 5) gastric wall changes revealed significantly higher IL-7 concentrations at T0 in dogs with moderate/severe gastric wall changes (*P* = 0.027) (Figure 2B). Concentrations of admission IL-6 and MCP-1 were also higher in dogs with moderate/severe gastric wall changes, but this difference did not reach statistical significance (both, *P* = 0.055) (Figures 2A,C).

## Discussion

In this present study we investigated the concentrations and kinetics of inflammatory cytokines, Ang-2, and CRP in dogs with GDV at 4 time points and compared them (except Ang-2) to plasma concentrations measured at a single time point in healthy control dogs. According to the inflammatory character of the disease, we expected a high concentration of pro-inflammatory biomarkers at admission, a further increase after systemic and gastro-intestinal reperfusion in the course of shock stabilization and gastric repositioning, and a decrease by 48 h after surgery. The study results show a diverse inflammatory profile of GDV dogs over a 48-h period with both pro- and anti-inflammatory patterns, with IL-6, IFN-γ, MCP-1, IL-10, and CRP being most involved in the inflammatory response. At admission (T0), 80% of dogs with GDV fulfilled the criteria for SIRS. An overlap of the clinical signs for SIRS with signs of cardiovascular shock is very likely, and both SIRS and shock are induced by and/or associated with the release of different inflammatory cytokines (1, 9). However, this was only marginally reflected by admission cytokine concentrations in our study, as IFN-γ was the only pro-inflammatory marker which was significantly increased compared to the heathy control group. It is possible that the inflammatory response was incompletely developed at this point, as GDV is a peracute disease. However, a recent study in dogs with GDV found significantly higher concentrations of cfDNA, HMGB-1, and PCT at admission compared with healthy control dogs (6), indicating detectable cell damage and inflammation early in the disease. Immediately after surgery (T1), concentrations of IL-6, and IL-10 were significantly increased compared to T0, and together with IFN-γ, significantly higher compared to the control group. General anesthesia and surgery, as well as systemic and local IRI due to fluid therapy and gastric repositioning are likely responsible for the exacerbated release of pro- and anti-inflammatory cytokines (4, 28–30). Fluid therapy may have diluted plasma levels of cytokines, as in this study dogs with GDV received a median fluid volume of 81 ml/kg over a median time period of 100 min between admission and start of surgery. The subsequent plasma dilution may have masked potential increases in other cytokines. At 24 h after surgery (T24), 67% of GDV dogs fulfilled SIRS criteria. At this time point, no overlap of clinical signs between cardiovascular shock and SIRS was expected since as cardiovascular shock should no longer be present. Four of the 11 pro-inflammatory markers, namely IL-6, IFN-γ, MCP-1, and CRP were significantly higher in dogs with GDV compared to control dogs, which coincides with the presence of SIRS. Again, surgical trauma as well as IRI are likely to be responsible for the clinical picture and cytokine profile. At 48 h after surgery (T48), the acute inflammatory reaction had subsided and only IFN-γ and CRP remained significantly higher in GDV dogs compared with control.

A key question in dogs with GVD is the prognosis and probability of gastric wall necrosis. Admission blood lactate concentration continues to appear to be a reliable marker to predict the presence of gastric wall necrosis with good sensitivity and specificity, low costs, and ubiquitous availability (31–34). Similarly, in the present study admission lactate concentrations were significantly higher in dogs with moderate/severe gastric wall changes compared to dogs with mild changes. In addition to lactate, in our study significantly higher admission plasma concentrations of IL-7 and a trend to a higher IL-6 and MCP-1 were found in dogs with a higher degree of gastric wall changes. However, the group of five dogs with moderate/severe gastric wall changes is too small and underpowered to draw a conclusion. To assess the importance and validity of these differences, an analysis in a larger group of dogs is necessary.

The interplay between the different cytokines is complex and the association of specific cytokines or cytokine groups with specific diseases and conditions is the subject of current research in human and veterinary medicine. Interleukin-6 is one of the major pro-inflammatory cytokines involved in the early acute phase reaction (35). Release of IL-6 is induced by several mechanisms, including tissue necrosis and presence of lipopolysaccharides, and it is a major stimulator of the synthesis of acute-phase proteins in the liver, such as CRP (21, 36). In dogs and other animal models, IL-6 increased within the 1st h after induction of sepsis and peaked after 4 h in dogs (37, 38). A significantly increased concentration of IL-6 as early as at the time of hospital admission was found in dogs with pancreatitis (39), non-infectious SIRS and sepsis (12), and trauma (16). A 2-fold increased concentrations of IL-6 were found at 6 h after laparotomy for ovariohysterectomy in healthy beagles, which decreased nearly to baseline at 18 h after surgery (40). In the present study, the concentration of IL-6 increased almost 5-fold after surgery (T1) and decreased to baseline 48 h after surgery. Apparently, this increase exceeds the purely surgical induced increase and is most likely multifactorial, including surgery, gastric reposition, and subsequent IRI. Monocyte chemotactic protein-1 is a chemokine, which is produced by a variety of cell types after induction by oxidative stress, cytokines, or growth factors, but monocyte/macrophages are found to be the major source (41). Significantly higher MCP-1 concentrations at hospital admission compared to healthy dogs were found in dogs with immune-mediated hemolytic anemia (IMHA) (10), sepsis (12, 13), trauma (16), and critical illness (13), amongst others. Further, studies in dogs have shown that a surgical procedure (e.g., laparotomy for ovariohysterectomy in healthy dogs) alone is not sufficient to significantly increase MCP-1 levels over a 30-h observation period (13, 40). An increase in MCP-1 at T1 and T24 in our study could therefore reflect IRI and systemic inflammation. Interferon-γ is produced primarily by T lymphocytes after contact with antigen-presenting macrophages and is characterized by its immunostimulatory, especially antiviral and antitumor effects. Among multiple other functions, it leads to the production of ROS, including hydrogen peroxide and nitric oxide, and is involved in the ischemia-reperfusion-induced leukocyte-endothelial cell adhesion (42). In our study, at T0 the concentration of IFN-γ was significantly higher compared to the control group and increased continuously until it reached the highest concentration at T48. In previous dog studies with trauma, surgery, sepsis, SIRS, IMHA, and neoplasia, IFN-γ was not found to be significantly changed (10, 15, 16, 37, 40, 43), although none of these studies evaluated this cytokine over 48 h. It is possible that this marker plays a more prominent role in states of oxidative stress and IRI than in other diseases not associated with reperfusion injury. The anti-inflammatory cytokine IL-10 inhibits the expression of pro-inflammatory cytokines, such as IL-6, and downregulates pro-inflammatory cytokine receptors (44). Concentrations are increased in humans and laboratory animals during endotoxemia, sepsis, and septic shock (45, 46). Various studies examining non-infectious SIRS revealed significantly higher IL-10 concentrations in dogs with IMHA (10), acute pancreatitis (39), and lymphoma (43), but no relevant increase was found in dogs with acute trauma despite a simultaneous pro-inflammatory profile (16). In the present study, dogs with GDV had significantly higher IL-10 concentration at all time points compared to healthy controls. This might represent a constant anti-inflammatory response induced early in the disease which lasts at least up to 48 h after surgery. Interleukin-10 was the only anti-inflammatory cytokine analyzed in this study; whether other anti-inflammatory cytokines would have reacted in the same way is unclear. Median concentrations of the pro-inflammatory IL-7, IL-8, IL-15, IL-18, and KC-like were below the concentrations of healthy control dogs at all time points in GDV dogs. Increased concentrations of these cytokines were found in different chronic, infectious and non-infectious inflammatory diseases in dogs (8), but they seem not to play an important role in our study group.

Angiopoietin-2 acts in both angiogenesis and inflammation (18), and can be released quickly following stimulation. It is highly expressed by endothelial cells at the infarct border zone after myocardial infarction in people or ischemia/reperfusion injury in mice and plays a role in post-ischemic cardiovascular remodeling and its deleterious effects (47). Furthermore, it is involved in the systemic inflammatory response that is stimulated by generalized infection, such as bacterial sepsis (18). Higher Ang-2 concentrations were found in dogs with SIRS and sepsis, and Ang-2 predicted negative outcome (19). In the present study, the authors expected an increase in Ang-2 concentrations due to potential endothelial activation and inflammation after IRI and surgery at T1 and T24. However, this was not reflected in the Ang-2 concentrations of our study dogs as the levels were relatively low at these times. Instead, Ang-2 seems to increase at 48 h after surgery. In a study evaluating kinetics of Ang-2 in poly-trauma patients, an increase in Ang-2 at days 4 and 7 was found in patients developing SIRS, but not in patients who did not developed SIRS (48). Likewise, in another study in septic pediatric patients, Ang-2 peak was at day 2 (49). It is important to note that unlike patients with GDV, patients with SIRS and/or sepsis usually have been sick for a certain period of time before admission. Therefore, the authors believe that Ang-2 increases too slowly to serve as a suitable marker for this hyperacute disease.

C-reactive protein is a major acute phase protein in dogs, which is known to increase after surgery (21). Canine serum CRP concentrations peaked at 12–24 h after abdominal surgery and then slowly declined toward baseline but remained elevated at 72 h (50, 51). Previous data indicate that in peracute conditions, such as GDV and trauma, the serum CRP concentration is initially normal, but increases during the 1st h of hospitalization (5). No difference in CRP concentrations has been found between surviving and non-surviving dogs with GDV or dogs undergoing invagination or partial gastrectomy, and its changes in concentrations were not rapid enough to serve as a preoperative prognostic marker in dogs with GDV (7, 23). The results of our study are consistent with previous findings. CRP levels peaked at 24 h and began to decrease, but remained elevated, at 48 h after surgery, indicating a delayed moderate inflammatory response.

We recognize that our study has limitations. The fluid therapy carried out during pre- and intraoperative stabilization certainly influenced the concentrations of inflammatory mediators in various ways in our study. Aggressive fluid therapy leads to hemodilution and potentially false low concentrations of different markers. Time T1, at which we expected the highest biomarker concentrations due to systemic and gastric reperfusion, was also the time point with the highest fluid volumes administered and thus potentially a high degree of hemodilution. A further difficulty for evaluation of the inflammatory reaction in our study is that fluid volumes during stabilization and hospitalization were not standardized but based on clinicians' preference and the patient's condition. Second, our control group was composed of healthy dogs and study markers were only determined at one time point. As mentioned before, surgery alone leads to increased concentrations of different inflammatory markers (13, 21, 40), and it is likely that the increase of these markers in our GDV dogs was at least partially due to the surgical procedure. A further limitation is the small group size, and a type I or type II error cannot be excluded. The group of non-survivors was too small to evaluate an association of biomarkers with outcome and a larger study group is necessary for analysis of this question. Lastly, while several statistical analyses were performed, a *post-hoc* correction for multiple comparisons, such as the Bonferroni correction, was not performed, as there is a risk of erroneously eliminating some of the reported results. This must be taken in consideration when interpreting our results.

In conclusion, the present study demonstrates that in dogs with GDV at admission only a mild pro-inflammatory-cytokine reaction was present. The pro-inflammatory reaction peaked immediately after surgery and up to 24 h post-surgery, whereby the pro-inflammatory response was mainly represented by IL-6, IFN-γ, MCP-1, and CRP, and decreased at T48 where only IFN-γ and CRP remained significantly increased. In addition, an anti-inflammatory response (based on the measurement of only IL-10) was found in GDV dogs at all time points.

## Data Availability Statement

The original contributions presented in the study are included in the article/supplementary material, further inquiries can be directed to the corresponding author.

## Ethics Statement

The animal study was reviewed and approved by Animal Experiment Committee of the Swiss Federal Veterinary Office (registration number BE 69/17). Written informed consent was obtained from the owners for the participation of their animals in this study.

## Author Contributions

AB assisted with study design, collected and analyzed data, and co-wrote the manuscript. AL assisted with study design and collected and analyzed data. SS and BH assisted with study design and edited the manuscript. LP and EM performed the laboratory analyses and edited the manuscript. K-NA designed the study, analyzed data, and co-wrote the manuscript. All authors contributed to, read, and approved the final manuscript.

## Conflict of Interest

The authors declare that the research was conducted in the absence of any commercial or financial relationships that could be construed as a potential conflict of interest.

## Abbreviations

Ang-2: angiopoietin-2
APPLE: acute patient physiologic and laboratory evaluation
BP: blood pressure
cfDNA: cell free DNA
CRI: constant rate infusion
CRP: C-reactive protein
ECG: electro-cardiogram
GDV: gastric-dilatation volvulus
HMGB-1: high mobility group box-1
IFN-γ: interferon-gamma
IL: interleukin
IMHA: immune-mediated hemolytic anemia
IV: intravenous
KC-like: keratinocyte chemotactic-like
MCP-1: monocyte chemotactic protein
PCT: procalcitonin
SIRS: systemic inflammatory response syndrome.
